# Identification of Unannotated Small Genes in *Salmonella*

**DOI:** 10.1534/g3.116.036939

**Published:** 2017-01-25

**Authors:** Jonghwan Baek, Jiyoung Lee, Kihoon Yoon, Hyunwoo Lee

**Affiliations:** * Department of Biopharmaceutical Sciences, University of Illinois at Chicago, Illinois 60607; † Next Sci Solutions, San Antonio, Texas 78256; ‡ Center for Biomolecular Sciences, College of Pharmacy, University of Illinois at Chicago, Illinois 60607

**Keywords:** genome annotation, ribosome profiling, small proteins, small genes, short ORF

## Abstract

Increasing evidence indicates that many, if not all, small genes encoding proteins ≤100 aa are missing in annotations of bacterial genomes currently available. To uncover unannotated small genes in the model bacterium *Salmonella enterica* Typhimurium 14028s, we used the genomic technique ribosome profiling, which provides a snapshot of all mRNAs being translated (translatome) in a given growth condition. For comprehensive identification of unannotated small genes, we obtained *Salmonella* translatomes from four different growth conditions: LB, MOPS rich defined medium, and two infection-relevant conditions low Mg^2+^ (10 µM) and low pH (5.8). To facilitate the identification of small genes, ribosome profiling data were analyzed in combination with *in silico* predicted putative open reading frames and transcriptome profiles. As a result, we uncovered 130 unannotated ORFs. Of them, 98% were small ORFs putatively encoding peptides/proteins ≤100 aa, and some of them were only expressed in the infection-relevant low Mg^2+^ and/or low pH condition. We validated the expression of 25 of these ORFs by western blot, including the smallest, which encodes a peptide of 7 aa residues. Our results suggest that many sequenced bacterial genomes are underannotated with regard to small genes and their gene annotations need to be revised.

Bacterial genomes serve as a blueprint in all aspects of biological research, and therefore accurate genome annotation is of paramount importance. However, increasing evidence indicates that currently annotated bacterial genomes have missed many genes encoding small proteins ≤60 aa (Wood *et al.* 2012; Warren *et al.* 2010). A small gene, or a small open reading frame (sORF), has previously been defined as one encoding proteins of ≤60 aa (Hemm *et al.* 2010); or alternatively, it accommodates those up to 100 aa (Andrews and Rothnagel 2014). While small proteins have been increasingly reported for their important cellular roles in bacteria (Alix and Blanc-Potard 2008; Martin *et al.* 2015; Hobbs *et al.* 2012), studies on small proteins are limited, partly because many small genes are unannotated in sequenced bacterial genomes (Alix and Blanc-Potard 2009; Storz *et al.* 2014). Despite much effort made to improve gene annotation, the accurate identification of small genes has been a persistent challenge (Warren *et al.* 2010; Wood *et al.* 2012).

Few experimental approaches are available that can complement the insensitivity of current annotation pipelines in detecting small genes. As noted in earlier studies (Rudd *et al.* 1998; Hemm *et al.* 2010), experimental approaches, such as mass spectrometry-based proteomics and two-dimensional (2D) gels, are not sufficiently sensitive to identify the majority of proteins whose size is <10 kDa. The most successful approach to identifying small genes, thus far, has been a targeted search for sORFs using *in silico* algorithms (Hemm *et al.* 2008; ÓhÉigeartaigh *et al.* 2014). In this approach, computationally predicted sORFs are sorted out based on the conservation of nucleotide/amino acid sequences in closely related bacteria and/or on the strength of ribosome binding sequences (RBS), thereby generating a list of candidate sORFs for experimental verification. A caveat of this approach is that species- (and strain-)specific sORFs and those without RBS may be missed. Moreover, simply because of short lengths of sORFs, a reasonable cut-off for conservation may not be obvious, and a low cut-off stringency can exponentially increase the number of candidate sORFs for experimental validation.

One promising experimental approach that allows for identification of sORFs is a genomic tool called ribosome profiling (Olexiouk and Menschaert 2016). In ribosome profiling, ribosome-protected mRNAs are sequenced, which provides a genome-wide snapshot of mRNAs being translated in cells grown in a given condition (Li *et al.* 2012; Oh *et al.* 2011; Ingolia *et al.* 2009). In this study, we applied the ribosome profiling to the Gram-negative pathogen *Salmonella enterica* Typhimurium 14028s grown in four different conditions. With the aid of *in silico* predicted putative ORFs (pORFs), we inspected the ribosome profiling data, and uncovered 130 unannotated ORFs. The majority of the unannotated ORFs identified encoded putative small peptides/proteins ≤100 aa. We also identified 139 genes that are incorrectly annotated. The results of our study represent the prevailing inaccuracy in the current bacterial genome annotations of small genes, and call for the development of a more robust annotation pipeline for better detection of small genes.

## Materials and Methods

### Strains and growth conditions

*S. enterica* serovar Typhimurium 14028s was the wild-type strain (Jarvik *et al.* 2010) used in this study. For ribosome profiling and mRNA sequencing experiments, wild-type cells were grown in Luria-Bertani (LB) medium (Sigma-Aldrich, Saint Louis, MO), MOPS EZ rich defined medium (RDM) (Teknova, Hollister, CA), modified N-minimal medium containing low Mg^2+^ (10 μM) and at pH 7.7, or modified N-minimal medium containing high Mg^2+^ (10 mM) and at low pH (pH 5.8) (Groisman *et al.* 1997). The overnight culture in LB or RDM was diluted 1000-fold into 200 ml of respective fresh media, and cells were grown at 37° to an optical density at 600 nm (OD_600_) of ∼0.4 and harvested by rapid filtration (Becker *et al.* 2013). The overnight culture in a modified N-minimal medium containing 10 mM Mg^2+^ and at pH 7.7 was washed twice with fresh N-minimal medium with no Mg^2+^ (pH 7.0) and diluted 100-fold into 200 ml of fresh N-minimal media at low Mg^2+^ concentration (10 μM) or low pH (pH 5.8). The cells in modified N-minimal media at low Mg^2+^ or low pH were grown at 37° to OD_600_ ∼0.3, respectively, and harvested by rapid filtration. For rapid filtration, a membrane of 0.45 μm pore size (Cat. No.: HPWP09050; Millipore, Billerica, MA) was used.

### Preparation of ribosome profiling and mRNA sequencing samples and Illumina sequencing

Ribosome profiling sequencing (ribo seq) samples were prepared as described previously (Oh *et al.* 2011) with a slight modification. Chloramphenicol was added to a final concentration of 0.4 mM in sucrose gradient solution for monosome isolation. Half of the cells harvested were used for isolation of total RNA, and mRNA sequencing (mRNA seq) samples were prepared as described previously (Oh *et al.* 2011; Becker *et al.* 2013) with a slight modification. Ribosomal RNAs were depleted by using the Ribo-Zero Magnetic Kit (Epicentre, Madison, WI) according to the manufacturer’s protocol. The prepared samples were sequenced using Illumina Hi-sequation 2000. Biotinylated oligonucleotides used for depletion of rRNA and tRNA are listed in (Supplemental Material, Table S1 in File S2), and sequencing statistics are shown in Table S2 in File S2.

### Ribosome profiling and mRNA sequencing data processing and generation of pORF lists

Both ribo seq and mRNA seq raw data were processed as described previously (Oh *et al.* 2011; Becker *et al.* 2013). For ribo seq data, sequencing reads were mapped to the *S*. Typhimurium 14028s genome (CP001363.1), with two mismatches allowed. Ribosome density was calculated using the algorithm described previously (Oh *et al.* 2011; Becker *et al.* 2013). For comparison of the four different ribosome profiling data sets, ribosome density for each nucleotide was normalized by the respective total number of mapped sequencing reads, and its value was displayed as ribosome density per million (rpm). For mRNA seq data, sequencing reads were processed in a similar manner, and the number of normalized sequencing reads was calculated for each nucleotide. Lists of pORFs with (pORF_RBS_) and without (pORF_noRBS_) RBS were generated with the nucleotide sequence of the genome of strain 14028s (and those of 10 other *S*. Typhimurium and *Escherichia coli* K-12 MG1655) using a custom-written perl script (File S1).

### Identification of unannotated ORFs

Text files of processed ribo seq and mRNA seq data, pORF list, and *Salmonella* genome annotation were uploaded to the genome browser MochiView (Homann and Johnson 2010) for visualization and manual inspection. For calculation of reads per kilo per million (RPKM) values for (un)annotated genes, CLC genomics workbench (ver 8.0; Qiagen) was used. To generate a list of unannotated ORFs, we applied an arbitrary cut-off of >10 RPKM in both ribo seq and mRNA seq data.

### Determination of conservation of unannotated ORFs

Conservation of unannotated ORFs identified was determined by BLAST searches in genomes of *S. bongori*, *S. enterica* subspecies (*S*. Paratyphi and *S*. Typhi), and other enteric bacteria (listed in Table S3File S2). A local BLAST (blastp and tblastn) was run as a plugin in Geneious R9.1 (Biomatters, Auckland, New Zealand), and EcoBlast in ecogene (Zhou and Rudd 2013) was also used. Proteins ≥13 aa were considered conserved in *Enterobacteriaceae* if their identity was ≥50% over the entire protein, and they were present in one or more bacterial species other than *Salmonella*. For peptides/proteins ≤12 aa, the cutoff for conservation used was ≥80% identity.

### Construction of SPA-tagged strains

Mutant strains each carrying a chromosomal sequential peptide affinity (SPA) tag at the C terminus of an ORF were constructed as described previously (Zeghouf *et al.* 2004). The SPA tag contains TEV cleavage site, calmodulin binding peptide, and the 3×FLAG epitope. The SPA tag, together with a kanamycin-resistance cassette, was PCR amplified using pJL148 as template and with a pair of primers each containing 40–45 nt homologous to the upstream or downstream flanking regions of the stop codon. All primers used for construction of SPA-tagged strains are listed in Table S4 in File S2. The resultant PCR product was used to transform *S*. Typhimurium 14028s carrying pKD46 for λ Red recombinase-mediated homologous recombination (Datsenko and Wanner 2000). The correct fusion of the SPA tag in each mutant was confirmed by PCR and sequencing. To construct a control strain expressing only SPA tag, the SPA tag was amplified using a pair of primers HP1352 and HP1353 (Table S4 in File S2), and cloned into pTrc99A (Amann *et al.* 1988) between *Nco*I and *Sal*I sites, and the resultant plasmid was used to transform the wild-type strain.

### Validation of expression of sORFs by western blot

The SPA-tagged strains were grown in the respective growth medium used in ribosome profiling experiments, and harvested. Whole cells were resuspended in tricine sample buffer (Bio-Rad, Hercules, CA) and heated at 95° for 10 min. The total protein (equivalent to the number of cells at OD_600_ 0.05) was separated on a 16.5% tricine gel (Bio-Rad) and transferred to a PVDF membrane (Bio-Rad) according to the manufacturer’s protocol. The SPA-tagged protein was detected using a monoclonal anti-FLAG M2-alkaline phosphatase-conjugated antibody (Sigma-Aldrich, Saint Louis, MO) and CDP Star chemiluminescent substrate (Sigma-Aldrich) according to the manufacturer’s protocol. The Novex sharp prestained protein standard (Novex, Carlsbad, CA) was used as a size marker.

### Supplemental material

File S1 contains the perl script. File S2 contains oligonucleotides used for the depletion of rRNA and tRNA in ribosome profiling (Table S1); sequencing statistics (Table S2), list of bacterial genomes used in BLAST search (Table S3); primers used to construct SPA-tagged mutant strains, cloning and sequencing (Table S4); length distribution of annotated proteins in 11 S. Typhimurium genomes (Table S5); list of genes annotated in 14028s but undetected by pORF_noRBS_ (Table S6); and comparison of numbers of annotated genes detected and undetected by pORF_RBS_ in 11 *S*. Typhimurium and *E. coli* K-12 MG1655 (Table S7 in File S2). File S3 contains supplementary text describing the lists of pORF_RBS_ and pORF_noRBS_. Table S8, Table S9, Table S10, Table S11, Table S12, and Table S13 are separate Excel files: list of strain 14028s annotated genes undetected by pORF_RBS_ (Table S8); list of 139 misannotated genes in strain 14028s (Table S9); list of 130 unannotated ORFs identified in strain 14028s (Table S10); list of sORFs unannotated in strain 14028s but previously identified and annotated in *E. coli* K-12 MG1655 (Table S11); conservation of *mia* ORFs in 11 *S*. Typhimurium strains (Table S12); and conservation of *mia* ORFs in non-*S*. Typhimurium Gram-negative enteric bacteria (Table S13).

### Data availability

Strains are available upon request. Code used to generate putative open reading frames is provided in File S1. Both raw and processed ribo-seq and mRNA-seq data are available at GEO with the accession number: GSE87871. 

## Results and Discussion

When this study started, the genomes of 11 *S. enterica* serovar Typhimurium strains were available in the GenBank database (Mather *et al.* 2013; Kröger *et al.* 2012; Richardson *et al.* 2011; Luo *et al.* 2011; Izumiya *et al.* 2011; Jarvik *et al.* 2010; Patterson *et al.* 2012; Kingsley *et al.* 2009; McClelland *et al.* 2001; Hooton *et al.* 2013; Hoffmann *et al.* 2013). To choose a model *S*. Typhimurium strain for our study, we analyzed 11 *S*. Typhimurium annotation files, which contain the list of currently annotated chromosomal genes encoding known and putative proteins. Comparison of the length distribution of the annotated genes among the *Salmonella* genomes revealed that the genome of strain 14028s is annotated with the largest number of small genes (encoding proteins ≤100 aa): 24% (1275 of 5312 total annotated genes) in strain 14028s *vs.* ∼10–13% (427–598/4452–4722) in the other 10 strains (Table S5 in File S2). Notably, the largest number of the total annotated genes in the genome of strain 14028s among the 11 *Salmonella* genomes was due to the larger number of small genes (Table S5 in File S2). The result of this analysis shows that the number of annotated small genes varies significantly between *S*. Typhimurium strains, and suggests that either the genome of strain 14028s is overannotated, or the genomes of other strains are underannotated with regard to small genes. We noted that, whereas the annotation of 10 other *S*. Typhimurium genomes relied mainly on gene prediction algorithms (Mather *et al.* 2013; Hooton *et al.* 2013; Hoffmann *et al.* 2013; Patterson *et al.* 2012; Kröger *et al.* 2012; Richardson *et al.* 2011; Luo *et al.* 2011; Izumiya *et al.* 2011; Kingsley *et al.* 2009; McClelland *et al.* 2001), the 14028s genome annotation also included all potential genes annotated in all available *Salmonella* genomes (Jarvik *et al.* 2010), which might explain the overannotation of 14028s genome. We chose the relatively overannotated strain 14028s as the model bacterium.

**Figure 1:**
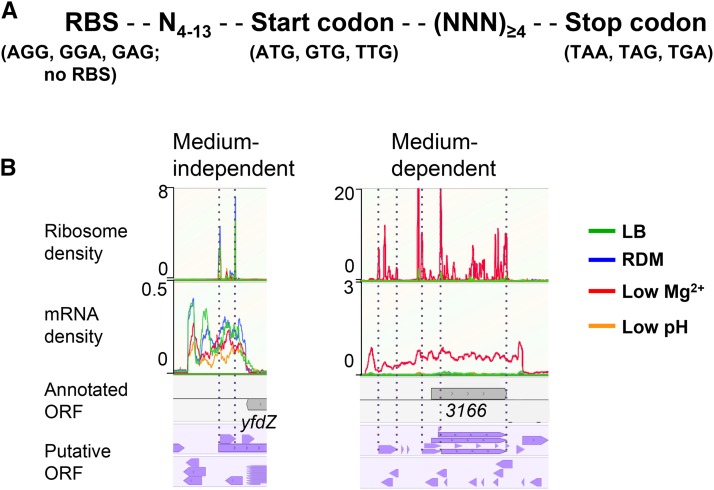
Identification of unannotated and misannotated ORFs. (A) Shown is the criteria of pORFs. Two different lists of *in silico* predicted ORFs were generated with the genome sequence of *S*. Typhimurium 14028s using a custom-written perl algorithm (File S1). (B) Visualization in the genome browser MochiView, and manual inspection of ribosome profiling and mRNA-seq data for identification of unannotated and misannotated ORFs. Shown are examples of three unannotated ORFs, one (medium independent) identified in all four growth conditions and two identified only in the low Mg^2+^ condition. In the example of “medium-dependent,” the annotated STM14_3166 (abbreviated as 3166) was identified as being misannotated; *y* axis represents the ribosome and mRNA density per nucleotide. Annotated genes and putative ORFs are shown in gray and purple arrow boxes, respectively.

To identify unannotated genes, we determined translatomes (ribo seq) and transcriptomes (mRNA seq) of strain 14028s cells grown in four different media: LB, RDM, N-minimal medium containing low Mg^2+^ (10 μM), and N-minimal medium at low pH (pH 5.8). Low Mg^2+^ and low pH are known to be host-mimicking conditions, in which *Salmonella* virulence genes, such as those required for survival within macrophages, are expressed (Kröger *et al.* 2013; Beuzón *et al.* 1999; García Véscovi *et al.* 1996). To facilitate the identification of unannotated genes, we generated two different lists of pORFs *in silico* with the nucleotide sequence of the genome of strain 14028s. One was called “pORF_RBS_” and the other “pORF_noRBS_” (Figure 1A). Detailed analysis of their utility is described in File S3. We also generated pORF_RBS_ lists with genomes of 10 other *S*. Typhimurium (and *E. coli* K-12) (Table S6 and Table S7 in File S2, and Table S8). Comparison between pORF_RBS_ lists of 14028s, and others, further indicated that the 14028s genome is overannotated with respect to small genes (File S3).

**Figure 2:**
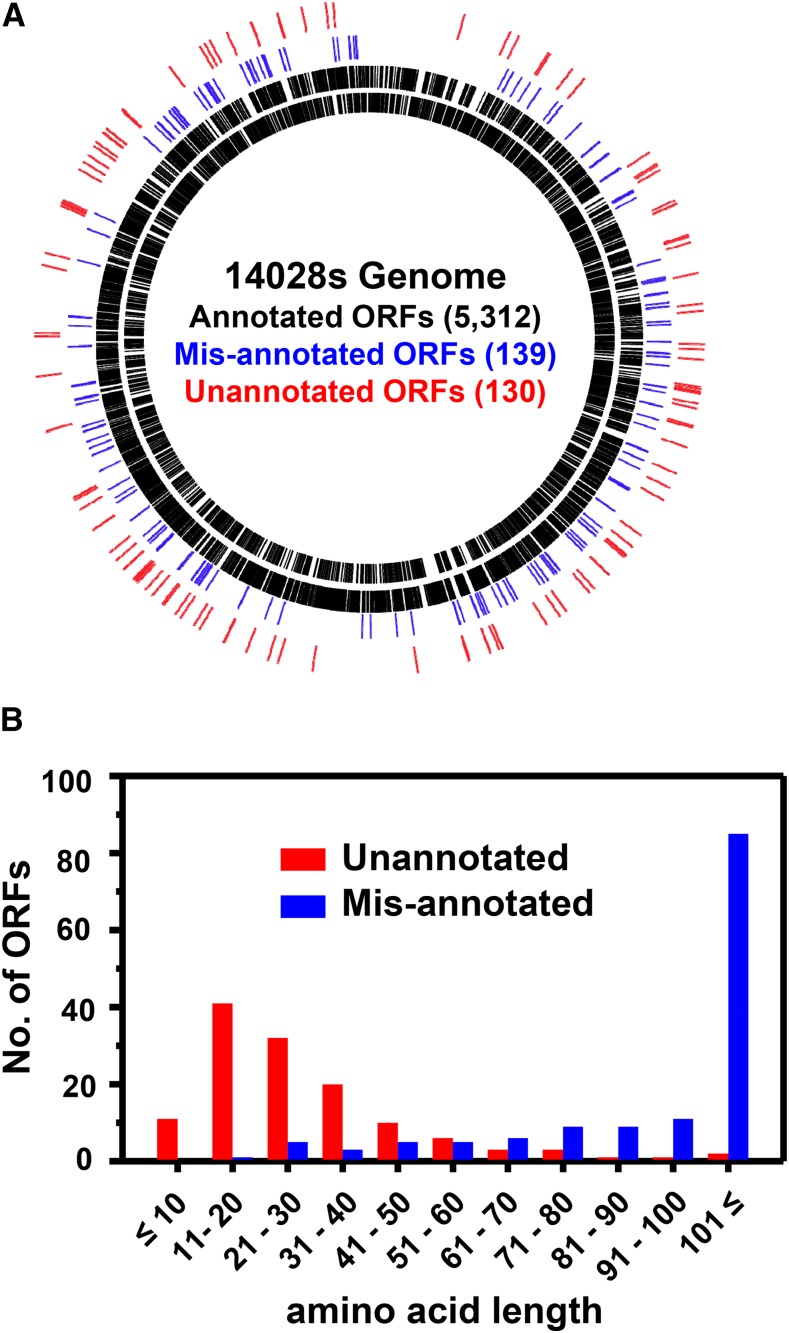
Genome-wide identification of misannotated and unannotated ORFs and their amino acid length distribution. (A) Misannotated (blue) and unannotated (red) ORFs identified were spread widely around the genome. (B) Unannotated ORFs were enriched with those putatively encoding small peptides/proteins ≤50 aa, whereas the majority of the misannotated genes encoded proteins >100 aa.

To search for unannotated ORFs, the ribo-seq and mRNA-seq data, in combination with the pORF list (either pORF_RBS_ or pORF_noRBS_), were uploaded to the Genome browser MochiView and were inspected manually. In most cases, ribosome density peaks for start and stop codons were readily distinguished due to their relatively high intensities as compared to those for most of the other codons in an ORF. Upon preliminary inspection of the ribo-seq data, we immediately noted that some gene annotations did not align with ribosome density and/or mRNA density due to their incorrect annotation (Figure S1), and, therefore, in addition to unannotated ORFs, we also attempted to find incorrectly annotated genes (called “misannotated”). As a result, we compiled 139 misannotated genes (Table S9) and 130 unannotated ORFs (Table S10). Some of the unannotated sORFs (Table S11) identified by this analysis were excluded from these lists because they had been previously identified (Hemm *et al.* 2008; Hobbs *et al.* 2012; Wong *et al.* 2000; Gaßel *et al.* 1999; Bishop *et al.* 1998) and annotated in the updated *E. coli* K-12 genome (Zhou and Rudd 2013), and previously characterized (MgtM, MgtP, and MgtR) in *S*. Typhimurium 14028s (Lee and Groisman 2012; Alix and Blanc‐Potard 2008). Misannotated genes and unannotated ORFs were designated “*man*-#” for *m*is*an*notation and “*mia*-#” for *m*issing *i*n *a*nnotation, respectively. The fact that *man* and *mia* were identified throughout the genome (Figure 2A) suggests that they are general problems occurring during the annotation process. Notably, the majority of the unannotated ORFs identified were sORFs (Figure 2B), as ∼87% (113/130) of them putatively encode peptides/proteins of ≤50 aa and ∼98% (128/130) of them ≤100 aa (Figure 2B). This was in sharp contrast with the misannotated genes, the majority of which encode proteins >100 aa (Figure 2B). These results clearly show that, despite the apparent overannotation of small genes in the 14028s genome as compared with 10 other *S*. Typhimurium genomes (Table S5 in File S2), many small genes are still missed during annotation, reflecting inaccurate small gene detection. The majority (114 of 130) of unannotated ORFs identified have apparent RBS with various strengths and spacing from corresponding start codons (Table S10). We examined the upstream nucleotide sequences of the remaining 16 unannotated ORFs without RBS; however, they did not show any common features (data not shown). Of 130 unannotated ORFs, 54% (70/130) have “ATG,” 28% (36/130) “GTG” and 18% (24/130) “TTG” as a start codon. While the ratio of ORFs with different start codons generally follows the trend reported for *E. coli* K-12 annotated genes (ATG:GTG:TTG = 83%:14%:3%) (Blattner *et al.* 1997), higher proportions of alternative start codons (GTG and TTG) may indicate that the accurate detection of genes with alternative start codons is more challenging in current annotation pipelines.

To determine the conservation of the *mia* ORFs identified, each ORF was BLAST searched in the genomes of *Salmonella* (10 *S*. Typhimurium strains, *S. bongori*, *S*. Paratyphi, *S*. Typhi) and other representative Gram-negative enteric bacteria (listed in Table S3 in File S2). Of the 130 *mia* ORFs, 53 were conserved in *Enterobacteriaceae*, 65 were specific to the genus *Salmonella*, and 12 could not be assigned due to their small ORF sizes (≤39 bp) (Table S12 and Table S13). *Salmonella*-specific ORFs could be further grouped into 28 ORFs that are present in both pathogenic (*S*. Typhimurium, *S*. Paratyphi, and *S*. Typhi) and nonpathogenic *Salmonella* (*S. bongori*); 22 only in pathogenic *Salmonella*; 11 specific in *S*. Typhimurium; three in *S*. Typhimurium strains and *S. bongori*; and one unique in *S*. Typhimurium 14028s. The discovery of unannotated sORFs in pathogenic and/or nonpathogenic *Salmonella* lays a foundation for their characterization in the context of *Salmonella* general physiology and pathogenesis.

**Figure 3:**
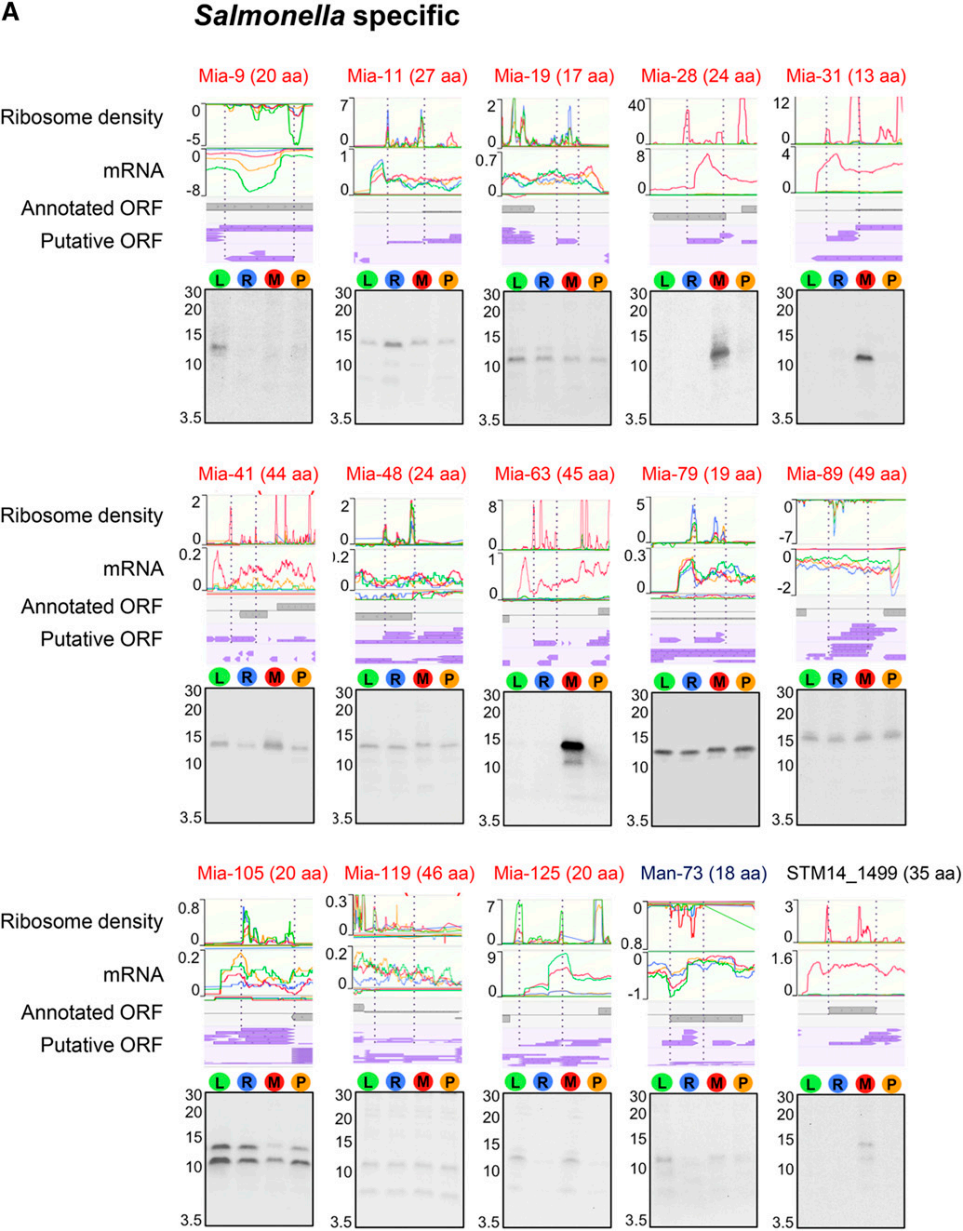

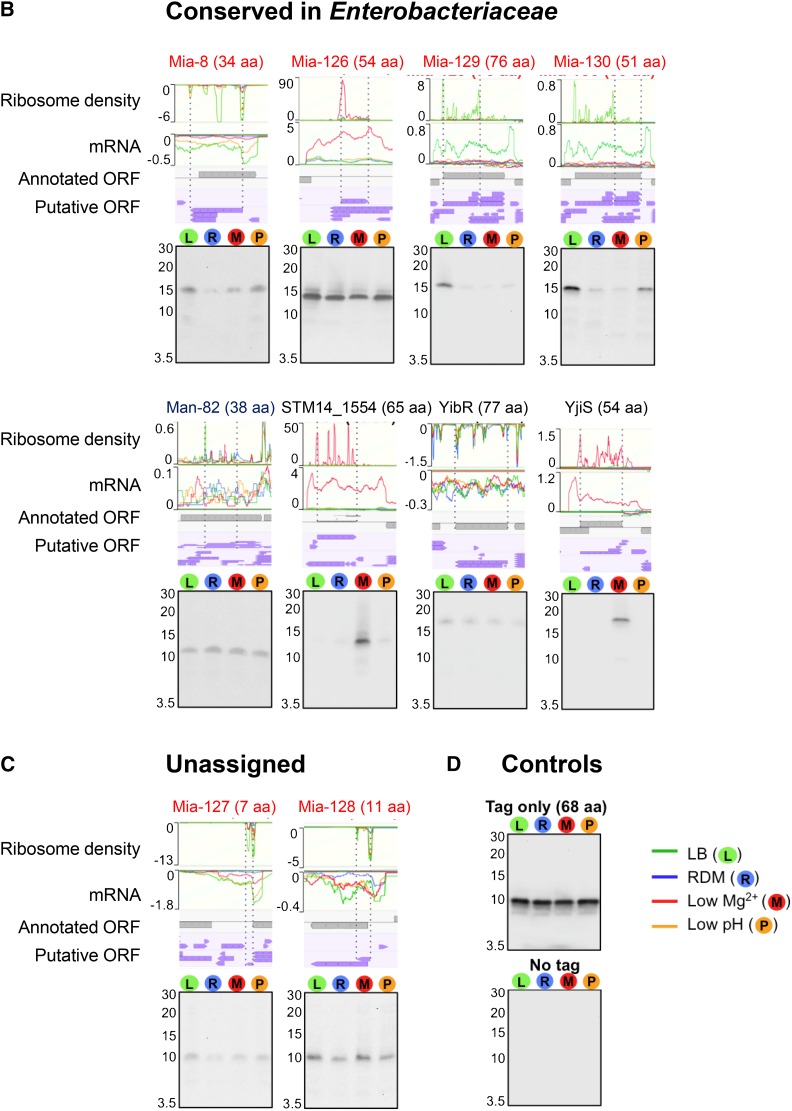
Verification of expression of selected misannotated and unannotated sORFs and small “*y*” genes by western blot. The sORFs and small genes examined for their expression are grouped into three categories: (A) *Salmonella*-specific, (B) conserved in *Enterobacteriaceae*, and (C) unassigned. “Unassigned” indicates sORFs whose conservation could not be determined by tBLASTn searches due to their short amino acids lengths. Mutant strains each carrying a chromosomal SPA tag fused to the C terminus of a target sORF/small gene were grown in respective medium used for ribosome profiling experiments (see *Materials and Methods*). Whole cell extracts (equivalent to the cell number at OD_600_ 0.05) were run on a 16.5% SDS-PAGE gel, and the expression of SPA-tagged peptides/proteins was determined by western blot using an alkaline phosphatase-conjugated anti-FLAG antibody. A negative value on the *y* axis (ribo or mRNA density) indicates genes are located on reverse strand. The positions of the markers are shown for the approximate sizes of proteins (kDa). As a negative and a positive control for western blot, the whole cell extracts of the wild-type (no SPA tag) and wild-type cells expressing only SPA tag (tag only) were used (D).

To validate the expression of unannotated, misannotated, and annotated sORFs identified by the ribosome profiling, we chose 27 sORFs (21 unannotated, two misannotated, and four annotated), which include those specific to *Salmonella* (Figure S2A), those conserved in *Enterobacteriaceae* (Figure S2B), and those whose conservation could not be determined due to their short lengths (Figure S2C). To determine their expression, respective sORFs were chromosomally fused to the SPA tag at their C-terminus, and their expression was examined by western blot. As controls, wild-type cells expressing only SPA tag (tag only) and wild-type cells (no tag) were used (Figure 3D). As a result, we confirmed the expression of 25 sORFs. The expression of two unannotated sORFs (*mia-6* and *mia-62*) could not be detected (data not shown); this could be either because their expression levels are too low, or because they are posttranslationally regulated and degraded in the tested conditions. The relative expression levels of most sORFs determined by western blot correlated well with their relative signal intensities in ribo-seq and mRNA-seq among different growth conditions. In the ribosome profiling data, several sORFs appeared to be expressed only in low Mg^2+^ conditions, and western blot results validated their condition-specific expression (Mia-28, Mia-31, and Mia-63 in Figure 3A; STM14_1554 and YjiS in Figure 3B), justifying the employment of different growth conditions for a more comprehensive identification of unannotated ORFs. Altogether, we verified the expression of 19 unannotated sORFs, as well as two misannotated and all four annotated small genes, demonstrating that most, if not all, of the unannotated sORFs identified from the ribosome profiling data are likely real protein-encoding genes.

In this study, we have reported the identification of misannotated and unannotated ORFs based on ribosome profiling data, with the aid of *in silico* predicted ORFs. The majority of the unannotated ORFs identified are small genes encoding proteins ≤100 aa. Other studies, in which the ribosome profiling was applied to *E. coli* (Oh *et al.* 2011), *Caulobacter crescentus* (Schrader *et al.* 2014), and *Staphylococcus aureus* (Davis *et al.* 2014), have reported inadvertent discovery of unannotated sORFs. Our findings add to the increasing recognition that current annotations of bacterial genomes have missed many small genes (Wood *et al.* 2012), and reflect the persistent problem of inaccuracy in detecting small genes, and in the curation of sequenced genomes (Keseler *et al.* 2014). Though we intentionally chose the *S*. Typhimurium 14028s genome annotated with the largest number of sORFs, we uncovered >100 unannotated sORFs, suggesting that other sequenced bacterial genomes are likely underannotated with regard to small genes. The results of our and other studies demonstrate the utility of ribosome profiling as a general and powerful experimental tool for finding small genes, and calls for consortial efforts to develop a more robust annotation pipeline that accurately detects small genes.

## Supplementary Material

Supplemental material is available online at www.g3journal.org/lookup/suppl/doi:10.1534/g3.116.036939/-/DC1.
