# Supplementary figures and images for: Identification of Unannotated Small Genes in Salmonella
Source: G3 (Bethesda). 2017 Jan 25;7(3):983–9. doi: 10.1534/g3.116.036939 (PMC5345727; doi:10.1534/g3.116.036939)

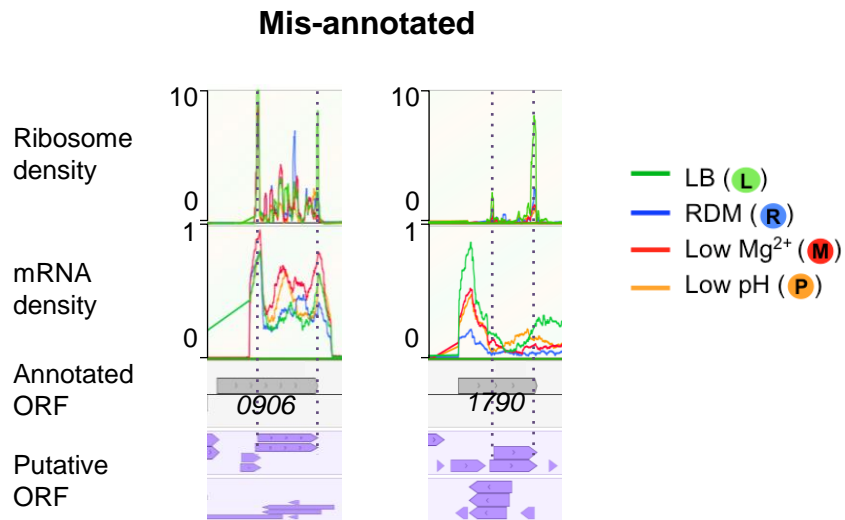

**Figure S1** Two examples of mis-annotated genes. STM14\_0906 and STM14\_1790 were identified as being mis-annotated.

Supplement: Supplementary file 1 [file 983FigureS1.pdf]
