# Supplementary material for: Identification of Unannotated Small Genes in Salmonella
Source: G3 (Bethesda). 2017 Jan 25;7(3):983–9. doi: 10.1534/g3.116.036939 (PMC5345727; doi:10.1534/g3.116.036939)
Supplement: Supplementary file 2 [file 983FigureS2.pdf]

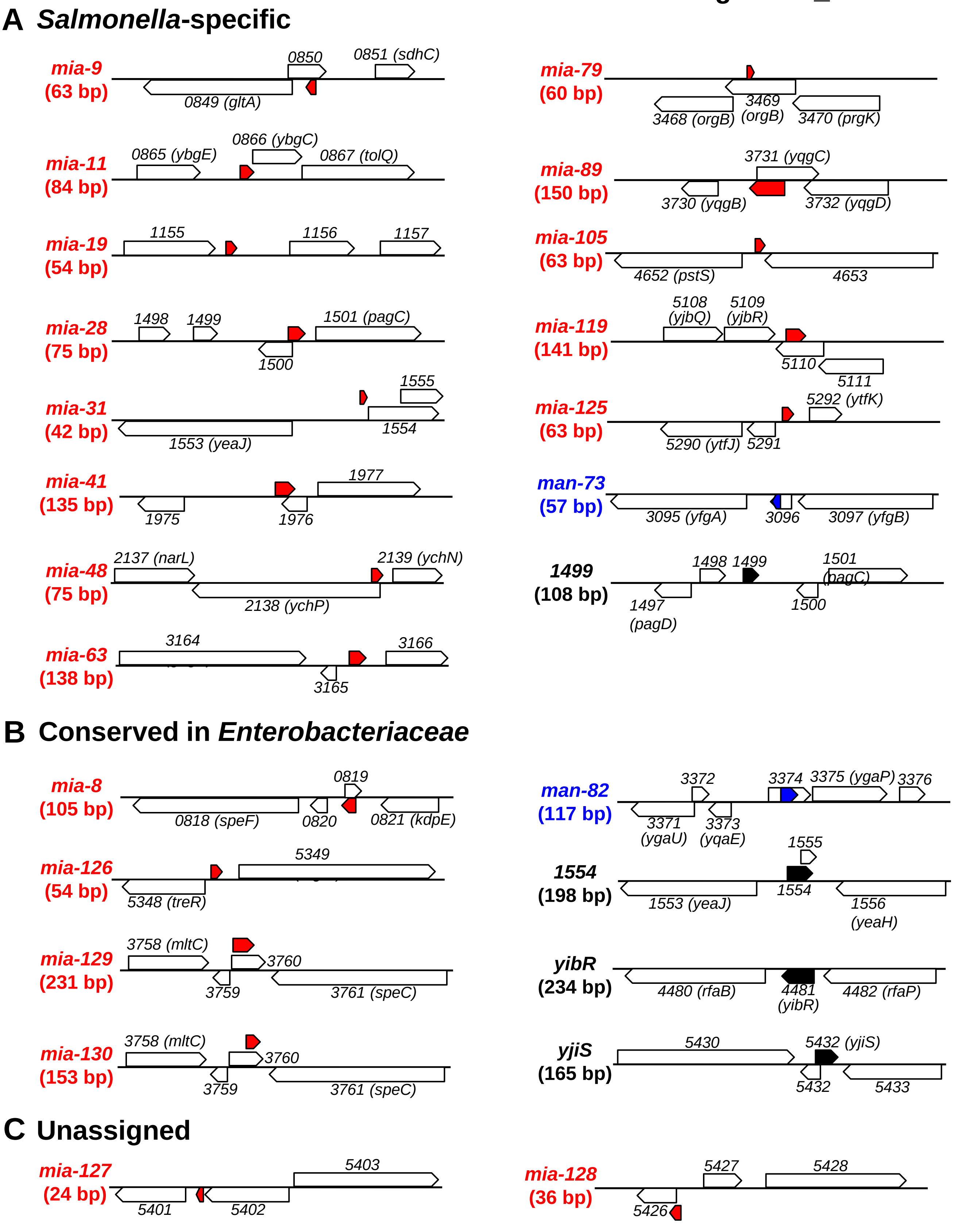

Figure S2 Genetic organization of (*mia* and *man*) sORFs and small annotated genes whose expression is validated by western blot.
