# Supplementary material for: Identification of Unannotated Small Genes in Salmonella
Source: G3 (Bethesda). 2017 Jan 25;7(3):983–9. doi: 10.1534/g3.116.036939 (PMC5345727; doi:10.1534/g3.116.036939)
Supplement: Supplementary file 4 [file 983FileS2.pdf]

Table S1 Oligonucleotides used for the depletion of rRNA and tRNA

Name	Nucleotide sequence
rRNA1	5'_/5Biosg[a]/TCATCTCCGGGGGTAGAGCACTGTTTCG_3'
rRNA2	5'_/5Biosg/GGCTAAACCATGCACCGAAGCTGCGGCAG_3'
rRNA3	5'_/5Biosg/AACACTGAGGCGTGACGACGAGGCACT_3'
rRNA4	5'_/5Biosg/CGGTGCTGAAGCAACAAATGCCCTGCTT_3'
rRNA5	5'_/5Biosg/CTCCTGATCGAAGCCCCGGTAAACGGCG_3'
rRNA6	5'_/5Biosg/CTTGCCCCGAGATGAGTTCTCCCTGAGA_3'
tRNA1	5'_/5Biosg/GGCGGTAACAGGGGTTCGAATCCCCTAG_3'
tRNA2	5'_/5Biosg/CCCCTTCGTCTAGAGGCCCAGGACACC_3'
tRNA3	5'_/5Biosg/GGACGTGGGGGTTCAAGTCCCCCCCTC_3'
tRNA4	5'_/5Biosg/GCGGTAGTTCAGTTGGTTAGAATACCT_3'

[a]5Biosg: Modification code in IDTDNA for 5′ Biotin.

Table S2 Deep-sequencing statistics of ribosome profiling and transcriptome experiments

Ribosome profiling (Ribo-seq)

Growth media	Total reads	Trimmed reads		Reads mapped to rRNA or tRNA		Reads mapped to genome			Reads mapped to pSLT plasmid			Unmapped Reads	
	No.	No.	%	No.	%	No	%	Coverage[a]	No.	%	Coverage	No.	%
LB	32,704,873	32,548,062	100	11,971,348	37	18,174,976	56	119	78,802	0.24	27	2,322,936	7
RDM	17,593,796	17,582,807	100	7,044,760	40	9,688,252	55	61	34,043	0.19	11	815,752	5
Low Mg^{2+}	31,420,973	31,412,150	100	17,358,286	55	13,669,502	44	89	100,291	0.32	34	284,071	1
Low pH	27,566,313	27,501,605	100	12,798,270	46	14,374,391	52	92	58,322	0.21	19	270,622	1

Transcriptome sequencing (mRNA-seq)

Growth media	Total reads	Trimmed reads		Reads mapped to rRNA or tRNA		Reads mapped to genome			Reads mapped to pSLT plasmid			Unmapped Reads	
	No.	No.	%	No.	%	No	%	Coverage[a]	No.	%	Coverage	No.	%
LB	28,132,254	28,122,883	100	22,685,518	81	3,841,521	14	25	28,059	0.10	10	1,567,722	6
RDM	34,893,678	34,885,425	100	27,652,407	79	5,412,931	16	35	27,375	0.08	9	1,792,712	5
Low Mg^{2+}	30,497,687	30,389,496	100	20,814,788	68	8,433,000	28	54	111,480	0.37	37	1,030,228	3
Low pH	27,377,904	27,313,213	100	18,092,550	66	8,106,101	30	54	84,303	0.31	29	1,030,259	4

[a]Coverage = (# of reads) X (average size of reads) / (genome or plasmid size)

Table S3 List of bacterial genomes used in BLAST search

Bacterial species	Strain	NCBI Genome/Nucleotide
Salmonella bongori	NCTC 12419	NC_015761.1
Salmonella enterica Typhimurium	LT2	AE006468.1
	T000240	AP011957.1
	4/74	CP002487.1
	UK-1	CP002614.1
	798	CP003386.1
	U288	CP003836.1
	CFSAN001921	CP006048.1
	D23580	FN424405.1
	SL1344	FQ312003.1
	DT104	HF937208.1
Salmonella enterica Paratyphi	ATCC 9150	NC_006511.1
Salmonella enterica Typhi	CT18	NC_003198.1
Escherichia coli	K-12 MG1655	U00096.3
	O157:H7 EC4115	NC_011353.1
	UTI89	NC_007946.1
Enterobacter cloacae	ATCC 13047	NC_014121.1
Serratia marcescens	WW4	NC_020211.1
Shigella dysenteriae	1617	CP006736.1
Shigella flexneri	2002017	NC_017328.1
Klebsiella oxytoca	E718	NC_018106.1
Klebsiella pneumoniae	342	NC_011283.1
Vibrio cholerae O1 biovar El Tor	N16961	NC_002505.1/NC_002506.1
Yersinia enterocolitica subsp. *palearctica*	Y11	NC_017564.1
Yersinia pestis	A1122	NC_017168.1

Table S4 Oligonucleotides used in this study

Name	Purpose	Nucleotide sequence (5′ → 3′)
HP1083	*mia*-8 SPA tagging	TGCCCACCGCAATAGTTTCGACTTTCATTTCTTTAATGCCCGGTCCATGGAAAAGAGAAG
HP1084	*mia*-8 SPA tagging	GGTCGGAAAGACCTGTATGCTGAAGTACCCGTAGCAGAAAAACTACATATGAATATCCTCCTTAG
HP1085	*mia*-8 sequencing	GCACGATATGCATAATCGCC
HP1112	STM14_5292 SPA tagging	TGAAGTTAACCGTCAGGTTATGCGTCTGCAAACTGAGATGGCGTCCATGGAAAAGAGAAG
HP1113	STM14_5292 SPA tagging	CGTTTTTTTTTACTTTTTTAACCTACTGCATAGCACTTTTGGTTACATATGAATATCCTCCTTAG
HP1114	STM14_5292 sequencing	GCGCCTTAAAACTGTTGGGC
HP1115	*mia*-79 SPA tagging	GGTAAAAATTCATTTAGGGCATTTAACTGTGCATACCCGACGCTCCATGGAAAAGAGAAG
HP1116	*mia*-79 SPA tagging	GTTTATCTGTCTGCAATAAGGCGCCGAATCACCGGTTTTTACTTACATATGAATATCCTCCTTAG
HP1117	*mia*-79 sequencing	CGCCTGCTGTTCTAATCTTT
HP1118	*mia*-126 SPA tagging	TGAACCCACCCCTCTCCCGCGATGGAGAATTTTCCTTTTCCGGTCCATGGAAAAGAGAAG
HP1119	*mia*-126 SPA tagging	TGTGTCACTGTCTTACACACCGGTAAGACAGCAGAGGCAGGCTTACATATGAATATCCTCCTTAG
HP1120	*mia*-126 sequencing	GGCGTGACGCTGATGGTGAT
HP1144	STM14_1829 SPA tagging	AGGTGGCCAAAATAGCCACAGTGGTGGACGGAAATCCGGCAATTCCATGGAAAAGAGAAG
HP1145	STM14_1829 SPA tagging	TGCAATATTTCAGAATATATTTATGAAATATCAGCAGTAAAATTACATATGAATATCCTCCTTAG
HP1146	STM14_1829 sequencing	CGCAACATCATCATGAGGAT
HP1147	*mia*-89 SPA tagging	CGGCGCTTCTCAGGATTCCAGAGCCGGCAATGGCGCTGAAGACTCCATGGAAAAGAGAAG
HP1148	*mia*-89 SPA tagging	GAAACCACGAGAAAACTCTTGCGTTAACAAGAGCGCCCTTGTTCACATATGAATATCCTCCTTAG

HP1149	*mia*-89 sequencing	GGCTTGTGGGTTCGTCTTAC
HP1150	*mia*-130 SPA tagging	CAAACACGTTCGCGTTCCGGCAAGTTCGCAACCCGTTGCACATTCCATGGAAAAGAGAAG
HP1151	*mia*-130 SPA tagging	AAGGCATTTACACCTTATCCGGCCTGCGAAACAGGGCAAGGATTACATATGAATATCCTCCTTAG
HP1152	*mia*-130 sequencing	CCGTCTTTATGCCGACCTTG
HP1153	STM14_4481 SPA tagging	CGCCTTAAAGCAAAAAGAGAGATGTCAGGTAAAAATTACTCTATCCATGGAAAAGAGAAG
HP1154	STM14_4481 SPA tagging	TAAAGACTTTAGATATTAAATAGCAAAAATAGTAATATAACCCTACATATGAATATCCTCCTTAG
HP1155	STM14_4481 sequencing	GAGTGAGACAAGGATAGACA
HP1156	*mia*-125 SPA tagging	CGTAGATATGGTCTATAGTCATATGGCATTAAAATTTGCGCCTTCCATGGAAAAGAGAAG
HP1157	*mia*-125 SPA tagging	AGAGTATTACGCCCTTGCGATGCCACAATCGGCCCAACAGTTTTACATATGAATATCCTCCTTAG
HP1158	*mia*-125 sequencing	GCCGATAGTTAATCATATGT
HP1219	*mia*-6 SPA tagging	CTTATGTAAGCGCCTGCAGAGTCGACGAGTTGCCGCCTTGATGTCCATGGAAAAGAGAAG
HP1220	*mia*-6 SPA tagging	GTTGTGCCGCCCTTCATTAGTATATACACAAAATCATTCGAGCTACATATGAATATCCTCCTTAG
HP1221	*mia*-6 sequencing	GGCGCATGAATGAACTTATG
HP1222	*mia*-9 SPA tagging	TTACTCGGATTGCTACGTTCCTGATTTGCTGCTAATTCGAAATTCCATGGAAAAGAGAAG
HP1223	*mia*-9 SPA tagging	ACCTGGGTACAGAGCAATGGGCGCTTGCAGGTAACGACTTGATTACATATGAATATCCTCCTTAG
HP1224	*mia*-9 sequencing	GTCGGGTTTTATCCTGAACA
HP1225	STM14_1449 SPA tagging	TGGAGCGTCACGTGAGGACTGTGAAGCACAATGCGATATGTTCTCCATGGAAAAGAGAAG
HP1226	STM14_1449 SPA tagging	CGTTCGGCTAAAAACATGTCATTAAGCAAACTCGCCATATAATCACATATGAATATCCTCCTTAG
HP1227	STM14_1449 sequencing	CGCGTGTTCAATATTTGCGT
HP1228	*mia*-28 SPA tagging	CATAGTGGCGATAACTGAATGCCGGATCGGTACTGCAGGTGTTTCCATGGAAAAGAGAAG
HP1229	*mia*-28 SPA tagging	TTCATAACAACTCCTTAATACTACTTATTATTTACGGTGTGTTTACATATGAATATCCTCCTTAG
HP1230	*mia*-28 sequencing	GCCGAACGGTGTCAAGTTTC

HP1231	*mia*-31 SPA tagging	TGAAGTGGTAAGGATAATAATGTACTTCCGTTCGGAGGCACTATCCATGGAAAAGAGAAG
HP1232	*mia*-31 SPA tagging	GCAATGCAAAAAGAAAAATTGTTGCCATTTCTTTAAATCGTTTCACATATGAATATCCTCCTTAG
HP1233	*mia*-31 sequencing	GTCGGATGCGTTAGTTTACC
HP1234	STM14_1554 SPA tagging	TTCTCTGCAATTACCGCCACCTCCTGGTGAGTATTGCTGGCATTCCATGGAAAAGAGAAG
HP1235	STM14_1554 SPA tagging	ACCTTACATGCGCCGACTTATCACTGAAATACTATGGATTTATTACATATGAATATCCTCCTTAG
HP1236	STM14_1554 sequencing	CGGCATCGATTTAACACTAA
HP1243	*mia*-128 SPA tagging	GAGGTGTGATGTGCTGATTGTTATCTGTAATCGCCACACGTTCTCCATGGAAAAGAGAAG
HP1244	*mia*-128 SPA tagging	CCGGTGAAAACACAGACAGGAAGAGACGCGTTCTGAATCTCGTCACATATGAATATCCTCCTTAG
HP1245	*mia*-128 sequencing	CCAGGATGCTGCGACTCTAC
HP1246	STM14_5432 SPA tagging	CGAGCAGTTAAGGGATATCGGGTTGGAAAGGAAGGATGTGGAATCCATGGAAAAGAGAAG
HP1247	STM14_5432 SPA tagging	ATCATAAAATCCTTTAATTACTATTGCCCTTGAATAGATTCACTACATATGAATATCCTCCTTAG
HP1248	STM14_5432 sequencing	CCACGTTGCACTAACCGTGC
HP1249	*mia*-129 SPA tagging	TGACCAATGGCTTCGGCTGTATCCTGGGCGGCATTGTGAGCGGTCCATGGAAAAGAGAAG
HP1250	*mia*-129 SPA tagging	TGCCAGTCGGTAATGCCGTTTTGAGTGTAATACTCCACCACTTTACATATGAATATCCTCCTTAG
HP1251	*mia*-129 sequencing	GCTGCAGCTTAAAATACTCT
HP1252	*mia*-105 SPA tagging	TGGCCCGGGGAGACACCAGACCAGCCTGCAGGGGGAGATGAATTCCATGGAAAAGAGAAG
HP1253	*mia*-105 SPA tagging	GATGTACGCGCTGTTGAAACCGTCTGCGGTTGCGCAAACAGTCTACATATGAATATCCTCCTTAG
HP1254	*mia*-105 sequencing	GCGACAACAGTTGCGACAGT
HP1255	*mia*-11 SPA tagging	TTACGGCGGCAAGCGGATGACGCCAACAAAGAGGCTGCAGGGATCCATGGAAAAGAGAAG
HP1256	*mia*-11 SPA tagging	CTTCGTAATAAACTCGAATTGGCCATCGAAACATATACTTATTCACATATGAATATCCTCCTTAG

HP1257	*mia*-11 sequencing	CCGCTCCTTGCGGATATCGT
HP1258	*mia*-19 SPA tagging	ATATGGAAATGTCGGAGCAGAACACCAAGAAGTAAGCGTAATTTCCATGGAAAAGAGAAG
HP1259	*mia*-19 SPA tagging	TTCAGATAAAAGAAAACCCGTACTCGGCGGGTTCACATTCATTCACATATGAATATCCTCCTTAG
HP1260	*mia*-19 sequencing	GGAGTTCAAGGATACCAGAG
HP1264	*mia*-48 SPA tagging	TCGGCAACTTCTACAACAAAAGATAAGAGGAATGACATCAGAGTCCATGGAAAAGAGAAG
HP1265	*mia*-48 SPA tagging	GCACGAGAAAAGGCTGCAAAACCTGTCCGGACACGTTATGGTTTACATATGAATATCCTCCTTAG
HP1266	*mia*-48 sequencing	CACCAGTATCCAGATCGTTA
HP1273	*mia*-62 SPA tagging	CCCAGGTAATTCTGTTCCCGTAACGCTCAACGTCCAGAGCAGGTCCATGGAAAAGAGAAG
HP1274	*mia*-62 SPA tagging	ATCTGAAGAAAAGAAAGCCGCCCAACAGGGCGGCCTCCTGGCTTACATATGAATATCCTCCTTAG
HP1275	*mia*-62 sequencing	CGGGAAACTATTCAATATTT
HP1276	*mia*-63 SPA tagging	GATAATACAAAACATTTTTCCAGTGGCTGGCCGCCAGATATCCTCCATGGAAAAGAGAAG
HP1277	*mia*-63 SPA tagging	CAGTATACTAACAACCACAAACCTGAGACCAATTCAGTTGCTTCACATATGAATATCCTCCTTAG
HP1278	*mia*-63 sequencing	GACAGTGAAGTGTGAACTAT
HP1279	*mia*-119 SPA tagging	AATAATCATATCGTCGTCGTCCAGCCAGCCGCGAGCATGTTCCTCCATGGAAAAGAGAAG
HP1280	*mia*-119 SPA tagging	CGACGCTATCCGCGAGTCCGCCGCGTTTATCGCCCGCGCGCTTCACATATGAATATCCTCCTTAG
HP1281	*mia*-119 sequencing	CCGGATTCACAACTCTATTA
HP1282	*mia*-127 SPA tagging	TTCAACTCAACCCGAGGCTTTGATGCTGGAGAATGTCATCATCTCCATGGAAAAGAGAAG
HP1283	*mia*-127 SPA tagging	ATTGATAATCAGTCCGGCCTGAAAAGGTCGGGTAACTGATTATCACATATGAATATCCTCCTTAG
HP1284	*mia*-127 sequencing	CGCAACAACCCTGTCAATGG
HP1285	*man*-8 SPA tagging	TATGTTTACGCGCTCATTGAACATCAGAGCAAATCAGACCGGCTCCATGGAAAAGAGAAG
HP1286	*man*-8 SPA tagging	ATGGCGGCAATGGCGTAACGCATCAGGCGAAAAGCCATCACATATGAATATCCTCCTTAG
HP1287	*man*-8 sequencing	CGCTGACAGAGTGGTCGCTA

HP1297	*mia*-41 SPA tagging	ATTCAATCCATTCCCTTTGATGATTCCTGTCAAACCTCCTGATTCCATGGAAAAGAGAAG
HP1298	*mia*-41 SPA tagging	GTACTTAACGTTATGTATTCCATCGCTGTTAACTAATTTACATATGAATATCCTCCTTAG
HP1299	*mia*-41 sequencing	GGCCCTGGATAGAGTGGTAA
HP1300	*man*-73 SPA tagging	AAGGCAGTATGTAATGGTGCAACAAGAGTTTAGCCTTGTGTCTTCCATGGAAAAGAGAAG
HP1301	*man*-73 SPA tagging	ACAGTATAAGCCTGTCAGGCCGCAGATGACAAAACCGCTACATATGAATATCCTCCTTAG
HP1302	*man*-73 sequencing	CGCGTGGCGATGACATTGAT
HP1303	*man*-82 SPA tagging	CGACGTCGCAACATCTGGCGCGGATGCGGGAAGAAGGGCTGATTCCATGGAAAAGAGAAG
HP1304	*man*-82 SPA tagging	AATGAAATAGTGAATGCGCTGGGCATCGCGTTGACTATCACATATGAATATCCTCCTTAG
HP1305	*man*-82 sequencing	CGCTGGCATTAGGTTGATGT
HP1344	pTrc99A sequencing	CGCTCAAGGCGCACTCCCGT
HP1352	SPA cloning into pTrc99A	AACATCCATGGAAAAGAGAAGATGGAAAA
HP1353	SPA cloning into pTrc99A	ACGACGTCGACCTACTTGTCATCGTCATCCTTGTAG

Table S5 Length distribution of annotated proteins in 11 *Salmonella enterica* serovar Typhimurium genomes

Salmonella strains	LT2	T000240	**14028s**	4/74	UK1	798	U288	CFSAN001921	D23580	SL1344	DT104
GenBank	AE006468	AP011957	**CP001363**	CP002487	CP002614	CP003386	CP003836	CP006048	FN424405	FQ312003	HF937208
≤ 50 aa	56 (1)[a]	127 (3)	**582 (11)**	179 (4)	70 (2)	50 (1)	133 (3)	120 (3)	46 (1)	59 (1)	51 (1)
51 aa – 100 aa	371 (8)	471 (10)	**693 (13)**	416 (9)	390 (9)	365 (8)	442 (10)	421 (9)	377 (8)	383 (9)	375 (8)
≤ 100 aa	427 (9.6)	598 (12.7)	**1275 (24)**	595 (12.9)	460 (10.3)	415 (9.6)	575 (12.6)	541 (11.9)	423 (9.5)	442 (9.9)	426 (9.5)
101 aa ≤	4025 (90)	4124 (87)	**4037 (76)**	4030 (87)	3995 (91)	3911 (90)	4006 (87)	4020 (88)	4023 (90)	4021 (90)	4070 (91)
Total	4452 (100)	4722 (100)	**5312 (100)**	4625 (100)	4455 (100)	4326 (100)	4581 (100)	4561 (100)	4446 (100)	4463 (100)	4496 (100)

[a]Number in parenthesis indicates percentage relative to the total number of annotated genes in respective genomes.

Table S6 List of genes annotated in 14028s but undetected by pORF$_{noRBS}$

Locus tag	Gene name	Description	Notes
STM14_980	ybiV(2)	putative hydrolase	[a]False annotation (?)
STM14_1180		minor tail protein	"CTG", unknown
STM14_1358		**pseudogene**	No start codon
STM14_1620	infC	translation initiation factor IF-3	"ATT", Known
STM14_1766	malX	**pseudogene**	No start codon
STM14_2252		putative cytoplasmic protein	[b]Mis-annotated
STM14_2277		putative cytoplasmic protein	[a]False annotation (?)
STM14_2439		hypothetical protein	[b]Mis-annotated
STM14_2450		portal protein	[b]Mis-annotated
STM14_3178		minor tail-like protein	[b]Mis-annotated
STM14_3265		**pseudogene**	No start codon
STM14_3349		**pseudogene**	No start codon
STM14_3673	prfB	peptide chain release factor 2	[b]Mis-annotated

STM14_3942		**pseudogene**, PTS family galactitol-specific enzyme IIC	No start codon
STM14_4405		**pseudogene**	No start codon
STM14_4596		**pseudogene**	No start codon
STM14_4676	yieN	regulatory ATPase RavA	[b]Mis-annotated
STM14_4709		putative inner membrane protein	[a]False annotation (?)
STM14_4714	rhoL	**pseudogene**	No start codon
STM14_4942		**pseudogene**	No start codon
STM14_4976		**pseudogene**	No start codon
STM14_5033		putative cytoplasmic protein	[b]Mis-annotated

[a]No sequencing reads were mapped to these annotated genes in both Ribo-seq and mRNA-seq; [b]Ribo-seq data indicate a new translation start codon for a corrected ORF.

Table S7 Number of annotated genes detected and undetected by $pORF_{RBS}$ in 11 *S*. Typhimurium and *E. coli* K-12 MG1655

	Genbank	Genome size (nt)	No. of predicted ORFs in $pORF_{RBS}$	Total annotated genes	No. of detected[a]	No. of undetected[b]	% undetected[c]	Annotated small genes (≤100 aa)	No. of annotated small genes undetected	% annotated small genes undetected
***S*. Typhimurium**										
UK1	CP002614	4,817,868	100,528	4,455	3,673	782	17.6	460	112	3
U288	CP003836	4,852,606	101,492	4,581	3,913	668	14.6	575	129	3
LT2	AE006468	4,857,432	101,564	4,452	3661	791	17.8	427	105	2
CFSAN	CP006048	4,859,931	101,681	4,561	3,814	747	16.4	541	134	3
**14028s**	**CP001363**	**4,870,265**	**101,767**	**5,312**	**3,952**	**1,360**	**25.6**	**1,275**	**670**	**13**
798	CP003386	4,876,219	101,877	4,326	3,120	1,206	27.9	415	146	3
SL1344	FQ312003	4,878,012	101,956	4,463	3,643	820	18.4	442	104	2
4/74	CP002487	4,878,013	101,956	4,625	3,506	1,119	24.2	595	235	5
D23580	FN424405	4,879,400	102,100	4,446	3,609	837	18.8	423	107	2
DT104	HF937208	4,933,631	103,567	4,496	3,589	907	20.2	426	118	3
T000240	AP011957	4,954,814	104,018	4,722	3,984	738	15.6	598	143	3
***E. coli* K-12**										
MG1655	U00096	4,641,652	103,026	4,140	3,597	543	13.1	423	44	1

[a] Number of annotated genes detected by $pORF_{RBS}$

[b] Number of annotated genes undetected by $pORF_{RBS}$

[c] % annotated genes undetected by $pORF_{RBS}$ (relative to the total number of annotated genes)
