# Supplementary material for: Identification of Unannotated Small Genes in Salmonella
Source: G3 (Bethesda). 2017 Jan 25;7(3):983–9. doi: 10.1534/g3.116.036939 (PMC5345727; doi:10.1534/g3.116.036939)
Supplement: Supplementary file 5 [file 983FileS3.pdf]

Supplementary Text

In both "$pORF_{RBS}$" and "$pORF_{noRBS}$" lists, pORFs were defined by any combination of canonical translation start codons (ATG, GTG, and TTG) and stop codons (TAA, TAG, and TGA), putatively encoding a peptide/protein of larger than or equal to 5 aa. The only difference between the two lists was that a pORF in "$pORF_{RBS}$" has a putative ribosome binding site. For a putative ribosome binding site, we considered any three consecutive nucleotides in the conserved ribosome binding sequence "AGGAGG" if they were located between 4 and 17 nucleotides upstream of a start codon (Figure 1A). As expected, the $pORF_{noRBS}$ list contained almost all of the 5,312 annotated genes of the 14028s genome, except 22 genes with non-ATG (-GTG or -TTG) start codons (Table S6). The genes with non-canonical start codons included the known *infC* gene with the ATT start codon, four unknown genes (STM14_980, STM14_1180, STM14_2277 and STM14_4709) with the CTG start codon, and seven genes that appear to be incorrectly annotated. The four genes with the CTG start codon appeared to be false annotations, because no sequencing reads were mapped to these annotated genes in the mRNA seq data (data not shown), and the remaining 10 genes were annotated as pseudogenes with no start codons (Table S6). By contrast, the $pORF_{RBS}$ list detected ~75% (3952/5372) of the total genes annotated in the genome of strain 14028s. Interestingly, of the annotated genes undetected by the $pORF_{RBS}$ list (1360/5372, ~25%), about half (670) of them were small genes (Table S7 and Table S8), indicating that many annotated small genes lack apparent ribosome binding sequences. For comparison, we also generated $pORF_{RBS}$ lists with genomes of other 10 *S.* Typhimurium (and *E. coli* K-12) strains and performed similar analyses (Table S7). The pORF numbers in other 10 *S*. Typhimurium and *E. coli* $pORF_{RBS}$ lists were exactly proportional to the nucleotide lengths of corresponding genomes and were similar to that of strain 14028s. However, the

numbers of annotated small genes that are detected and undetected by respective $pORF_{RBS}$ lists were significantly different. In strain 14028s, the number of undetected small genes was 670, which was much larger than those (104~235) in other 10 *Salmonella* and *E. coli* strains (Table S7). It was also notable that genes annotated in strain 1408s but undetected by $pORF_{RBS}$ were enriched with small genes (Table S8), many of which showed no or extremely low expression levels in the growth conditions tested, perhaps suggesting that many of these annotated small genes are not real. Taken together, the results of these analyses further indicate that the 14028s genome is over-annotated with respect to small genes, either with or without an apparent ribosome binding site, and reveal a prevailing inconsistency in the annotation of small genes among 11 *S*. Typhimurium genomes.
